# Plasticity of Bead-on-Plate Welds Made with the Use of Stored Flux-Cored Wires for Offshore Applications

**DOI:** 10.3390/ma13173888

**Published:** 2020-09-03

**Authors:** Aleksandra Świerczyńska, Michał Landowski

**Affiliations:** Faculty of Mechanical Engineering, Gdańsk University of Technology, 80-233 Gdańsk, Poland; miclando@pg.edu.pl

**Keywords:** flux-cored arc welding, welding wire, storage conditions, plasticity, diffusible hydrogen, atmospheric corrosion

## Abstract

Extreme atmospheric conditions in the marine and offshore industry are harmful to engineering materials, especially to welded joints, and may cause degradation of their properties. This article presents the results of research on the plasticity of bead-on-plate welds made using two types of seamless, copper plated flux-cored wires. Before welding, spools with wire were stored for 1 month in two distinct locations with different geographical and industrial conditions in Poland, and then subjected to visual examination. Bead-on-plate welds were subjected to a static tensile test and on this basis plasticity indexes showing the effect of storage on plasticity were determined. The fractures after tensile tests and the surfaces of the wires were examined on an electron scanning microscope. Additionally, diffusible hydrogen content in deposited metal measurements for each condition were carried out. The highest degradation level was found for wire stored in an agricultural building in north-eastern Poland—there was an almost fourfold decrease in the plasticity index value and the highest diffusible hydrogen content. For the same wire and the same location, the largest difference was also observed in fracture morphology after the tensile test—ductile fracture was obtained for wire at delivery condition while an almost full cleavage fracture was found after relatively short (1 month) storage of wire.

## 1. Introduction

Predicting environmental loading is one of the key challenges in the design and construction of offshore structures [1,2,3]. These harmful loads include the influence of wind and water: waves, ocean currents, and even atmospheric precipitation [4,5,6]. In difficult marine conditions, in addition to mechanical static loads, material fatigue and environmental destruction mechanisms play a decisive role in assessing structural durability [7,8]. According to the “DNV-RP-C201 Recommended Practice—Environmental Conditions and Environmental Loads” standard [9], environmental conditions affect design and technological decisions on which the life of the structure depends. This applies to the properties of the materials used and their joining technologies, including welding processes [10,11,12,13,14].

One of the welding processes commonly used in the production of ocean engineering and marine constructions is flux-cored arc welding (FCAW, 136 process acc. to ISO 4063:2009 standard). During the FCAW process, flux-cored wires are used as the electrode and at the same time as the consumable [15,16,17]. They are made of a metal sheath filled with ceramic or metallic powder. With such a structure, it is possible to achieve a favorable spray metal transfer in the welding arc, higher wire melting rates and the use of the core of wire for metallurgical purposes [18,19]. The consumption of flux-cored wires for ocean and marine structures is still increasing. This is the result of many unquestionable advantages of the FCAW welding process: high welding efficiency, penetration depth, good arc stability and relative ease of training of welders [15,20,21,22].

Many FCAW-welded structures can be classified as responsible structures, with a high risk of suffering significant economic and health losses in the event of failure or destruction. In manufacturing practice, flux-cored wires are often used in extreme marine and offshore conditions, mines and underground construction sites: tunnels and subways, in climatic zones with high temperature and relative humidity. This means that the quality control of the welding process should be at a high level, including before, during and after welding control. The procedure of producing flux-cored wires is more complicated than solid wires and requires closing the sheath, which is accomplished by bending its edges—for wires with the seam or by welding—for seamless wires. The quality of the wires and the stability of the arc also depend on the winding of the wire on the spool [23]. An important issue in this respect is also the type of spool (plastic or welded from steel wires), the type of wire packaging (e.g., cardboard, with additional aluminum or plastic foil, sometimes hermetic) and the use of moisture absorbing agents, as well as appropriate storage in warehouses and transport (e.g., on pallets, applying restrictions on the number of layers of packaging). Regardless of the method of production, consumables must be stored under controlled temperature and humidity conditions in order to preserve their declared properties [20,24]. Manufacturers specify ranges of these condition values which are safe from the point of view of the possibility of making sound joints. Their permissible values depend on the type of consumable and are usually equal to: temperature = 15–30 °C, relative humidity = max 70%. In welding practice, this aspect of pre-welding inspection is often neglected and materials are exposed to adverse environmental conditions. Even assuming proper supervision of transport, warehousing and storage environmental influence on the wires is difficult to avoid. This is a threat especially in the extreme conditions prevailing in the maritime industry characterized by high humidity, the presence of salt and often variable temperature values [20,25,26].

Dirt and moisture inside and on the surface of the flux-cored wire can get into the welding pool and later into all welded joint zones during welding. This can result in a reduction in the performance of such a welded joint or even its complete destruction. The literature describes such cases in the form of, among others, hydrogen embrittlement, various types of corrosion phenomena, cold or hot cracking [27,28,29,30,31,32]. Especially just after welding, when there may be (due to incorrect storage of wires) a large diffusible hydrogen amount inside the joint, changes in properties can be very dangerous. The impact of remaining stress on a brittle welded joint structure saturated by a critical diffusible hydrogen amount can lead to cold cracking [33,34,35].

A feature of the metal, which well describes its applicability, in addition to strength and toughness, is plasticity. It can be described by various factors, among others by elongation, deformation or narrowing. To determine the plasticity of the material, a static tensile test is carried out on smooth specimens. Studies have also been carried out on the impact of notch on the strength properties of metals. They show that the increase in elastic stress concentration factor is a cause for the increase in both the yield and tensile strength [36]. At the same time, the increase in stress triaxiality leads to a reduction of plastic strain at failure, which means that not only the presence of the notch but also its dimensions strongly affect plasticity [37]. This relationship means that the results of static tensile tests of notched specimens cannot be related to typical properties of the tested material. In contrast, the specimens with the notch can be compared with each other and provide information, e.g., on the effect of factors, other than the presence of the notch, on the specimen. Świerczyńska [20] conducted studies about properties of padding welds made using flux-cored wires stored in two different environments. After one month of storage in real, urban, outdoor conditions, significant changes in ultimate tensile strength tested with the intrinsic hydrogenation were found.

Despite the great practical significance of problems associated with the storage of welding consumables, this topic is relatively rarely discussed in the literature. Previous works mainly concerned the impact of wire construction and storage conditions on the diffusible hydrogen amount in weld metal [18,22,38] and the influence of welding parameters and conditions on the quality and properties of welded joints [20,39,40,41,42]. Accordingly, the purpose of the work was to analyze the impact of storage conditions of seamless, copper plated flux-cored wires on the plasticity of the bead-on-plate welds made with these wires.

## 2. Materials and Methods 

In this study, rutile flux-cored wires with the designation T 46 2 P M21 2 H5 (according to EN ISO 17632-A standard) were used. For comparison purposes, two wire grades with similar structures made by different manufacturers were selected: seamless, copper plated, with 1.2 mm diameter, both intended for making welded joints for offshore and marine structures. For the purposes of this article, the wires are designated as “A wire” and “B wire”. They were stored in open packaging in real, uncontrolled, measured conditions, in which temperature and relative humidity were measured every 30 min to recognize how those conditions changed with time. Those changes were recorded in situ using a recorder Termioplus (Termoprodukt, Bielawa, Poland) with SHT15 sensor (temperature recording with a resolution of 0.1 °C, humidity registration with a resolution of 0.1% RH). Locations of storage were: welding site in Gdańsk (Gdańsk welding site—GWS) (54°20′51″ N 18°38′43″ E)—a Baltic Sea maritime economy center in north Poland with a large commercial port, and agricultural building in Mońki (Mońki agricultural building—MAB) (53°24′25″ N 22°47′49″ E)—a small town in north-eastern Poland with food enterprises. Those locations were chosen to compare different geographical and industrial conditions and their impact on wires. The wires were stored without packaging, without direct contact with precipitation, the welding site was heated from the urban heating system and the agricultural building was not. After 1 month of storage (March-April) in each location, wires were visually tested and used for welding. The results of the measurements of storage conditions were described statistically using values: minimum, maximum, range, mean, standard deviation and the coefficient of variation. Coefficient of variation (*V*) was calculated using the formula:

(1) $$V=\frac{S}{X}\times 100\%$$

where *S* is the standard deviation and *X* is the mean value. An increase in the coefficient value indicates a greater variability of the described parameter. 

A Hung Ta HT-2401 (Hung Ta Instrument Co., Ltd., Taichung, Taiwan) testing machine with a deformation speed of 2 mm/min was used to check the basic mechanical properties of base material used for the study. Five specimens were subjected to the test and mean values are given.

**Figure 1:**
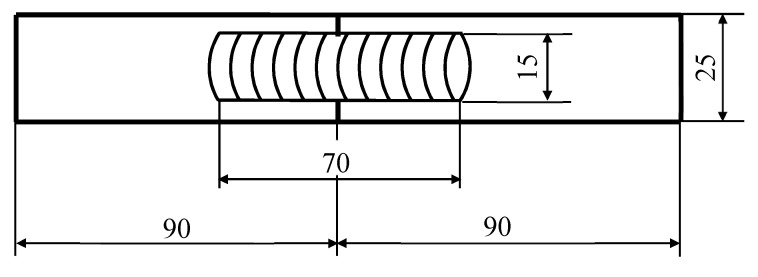
Scheme of test specimen and dimensions in millimeters.

Bead-on-plate welds were made on S355J2 steel specimens with thicknesses of 10 mm. Dimensions of specimens are given in Figure 1, chemical composition of base material and weld metal made with the use of wires is given in Table 1. Basic mechanical properties of weld metal made with the use of wires are given in Table 2.

Welding was performed on the robotized station ABB IRB 2400 with Migatronic BDH320 (Migatronic A/S, Fjerritslev, Denmark) welding power source. Welding was carried out at a mean welding current of 210 A, arc voltage 25 V, welding speed 30 mm/min, stick out length 20 mm and shielding gas (mixture Ar + 18% CO_2_, M21) flow rate 17 L/min. The bead-on-plate welds were about 70 mm long. Immediately after welding, the specimens were cooled in water and put into liquid nitrogen to inhibit hydrogen diffusion. The tensile tests of the bead-on-plate welds brought to ambient temperature were carried out on a Hung Ta HT-2401 (Hung Ta Instrument Co., Ltd., Taichung, Taiwan) testing machine with deformation speed 2 mm/min. Three trials were performed for each condition.

Since the specimens were not smooth, it was impossible to use standard plasticity measures. To compare individual specimens and the influence of conditions in which they were stored, our own measure of relative elongation was used:

(2) $$E_{r}=\frac{E_{p_{0}}}{E_{p_{1}}}$$

where:

*E_r_*—plasticity index;
*E_p_*_1_—mean plastic elongation of specimens made using stored wire (mm);
*E_p_*_0_—mean plastic elongation of specimens made using wire in delivery condition (mm).

Plastic elongation was determined from the tensile test stress–strain curves. Elongation was determined by indicating the total strain to fracture and then by determining the range of elastic deformation from Hook’s law, the plastic elongation was calculated.

The scanning electron microscope (SEM) JEOL JSM-7800F (Japan Electron Optics Laboratory Co., Ltd., Tokyo, Japan) was used for fracture observations of specimens after the tensile test and for observations of wire surfaces. Spot tests of the chemical composition in selected areas of the wires’ surfaces were also carried out with the use of EDAX adapter enabling EDS analysis.

Specimens after the test were subjected to fracture observations using the scanning electron microscope (SEM) JEOL JSM-7800F (Japan Electron Optics Laboratory Co., Ltd., Tokyo, Japan).

Diffusible hydrogen content in deposited metal (H_D_) was measured using the mercury method in accordance with EN ISO 3690 standard [31]. The specimens were made using the same welding parameters, the same tooling and the same equipment as bead-on-plate welds for tensile tests. Three specimens were made for each condition with the same dimensions of the welds and the arithmetic mean was taken as a result.

## 3. Results

**Figure 2:**
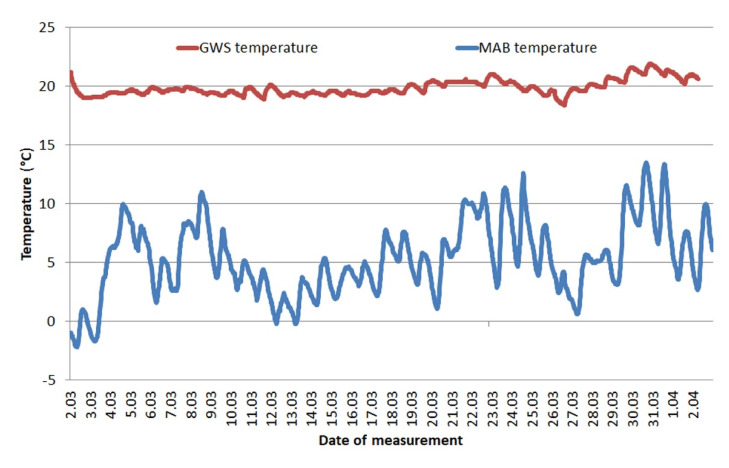
Ambient temperature changes during wires storage—Gdańsk welding site (GWS) and Mońki agricultural building (MAB)—1 month.

A comparison of temperature changes in GWS and in MAB is shown in the Figure 2. In addition, theTable 3 presents values of statistical measures and coefficients describing the temperature changes at each location in a given time period.

A clear difference is visible between the temperatures in MAB and GWS. For MAB, the temperature curve is always below the temperature curve measured in GWS. Storage conditions in GWS were stable in terms of temperature, temperature shocks are not visible.

**Figure 3:**
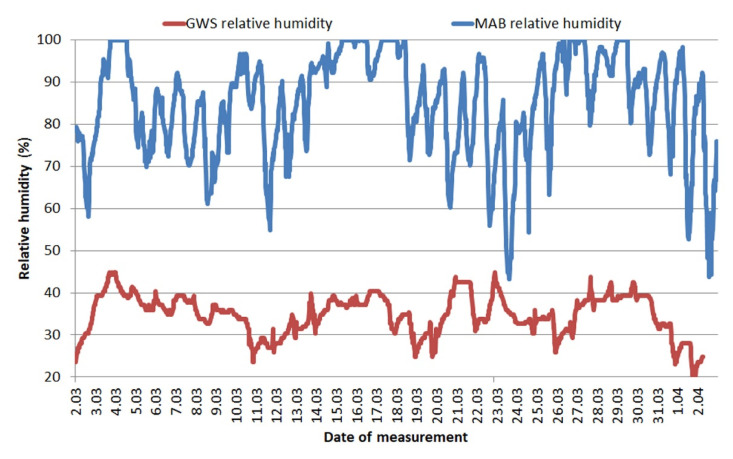
Ambient humidity changes during wires storage—Gdańsk welding site (GWS) and Mońki agricultural building (MAB)—1 month.

The Figure 3 shows a comparison of changes in relative humidity at the same time and for the same places. Coefficient values describing the change in this property are also given in Table 3.

For MAB, the registered humidity is very high, reaching up to 100%, which means that in the place where the wires were stored, the air was saturated with water vapor. The humidity was clearly lower in GWS. It did not exceed 50% at any time. Differences in the waveforms of temperature and relative humidity result, among others, from the fact that the GWS was heated.

The calculations of the factors describing the conditions confirm the observations of the curves. The lowest temperature recorded was −2.2 °C in MAB, while the highest was recorded in the GWS and it reached 21.9 °C. The standard deviation of the mean is very important, it indicates how stable the conditions were in each location. As can be seen, GWS shows much greater temperature stability—the range of temperature results over a period of 1 month was only 3.5 °C and standard deviation was 0.7. At the same time, the second location (MAB) was exposed to very large temperature differences—the range of results was up to 15.7 °C. The coefficients describing relative humidity in MAB clearly show how unfavorable conditions persist there—a mean value of 85% with a deviation of 12.1 clearly shows the persistence of extreme conditions for flux-cored wires. The quantitative assessment of the conditions in the GWS confirms that despite the greater variability of humidity than temperature, throughout the storage period manufacturers’ recommendations regarding maximum relative humidity (max 70%) have not been exceeded.

**Figure 4:**
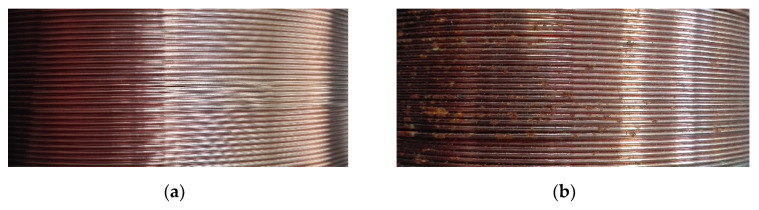

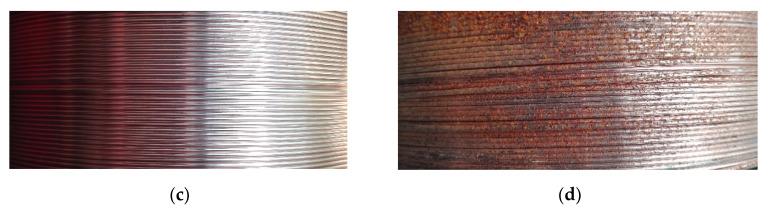
The surface layer of wire after storage: (**a**) A wire stored in Gdańsk welding site (GWS); (**b**) A wire stored in Mońki agricultural building (MAB); (**c**) B wire stored in Gdańsk welding site (GWS); (**d**) B wire stored in Mońki agricultural building (MAB).

Wires in delivery condition and wires stored for 1 month have been subjected to visual examinations. Photographs of the surface layer of those wires in delivery condition were already presented in the previous publication [20]. Pictures of the surface of the stored wires are shown in Figure 4. It can be clearly seen that the surface of wires stored in MAB has greater degradation than the surface of wires stored in GWS.

Differences can also be seen between the degradation that occurred in A wire and B wire. Both wires stored in the GWS have a shiny, smooth surface with no corrosion areas and no visible signs of degradation or environmental impact. In the MAB environment, A wire began to undergo surface corrosion—numerous corrosion areas of a small range are visible. The boundaries between the individual coils are clear, the non-corroded areas are still shiny and smooth. The B wire stored in the same environment was significantly more degraded. The entire surface is covered with corrosion products, which in some places have a significant thickness. Areas without a thick layer of rust were covered with a thin layer of corrosion products and there were no shiny areas.

**Figure 5:**
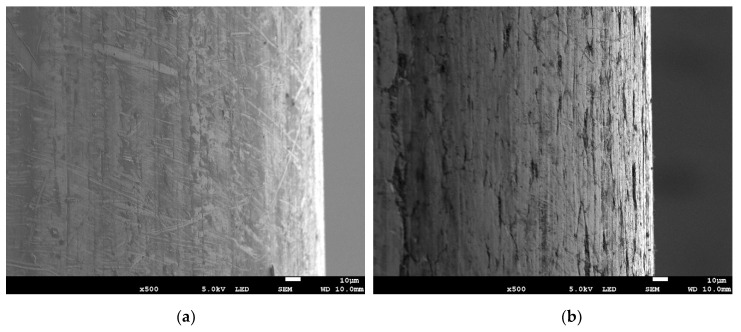

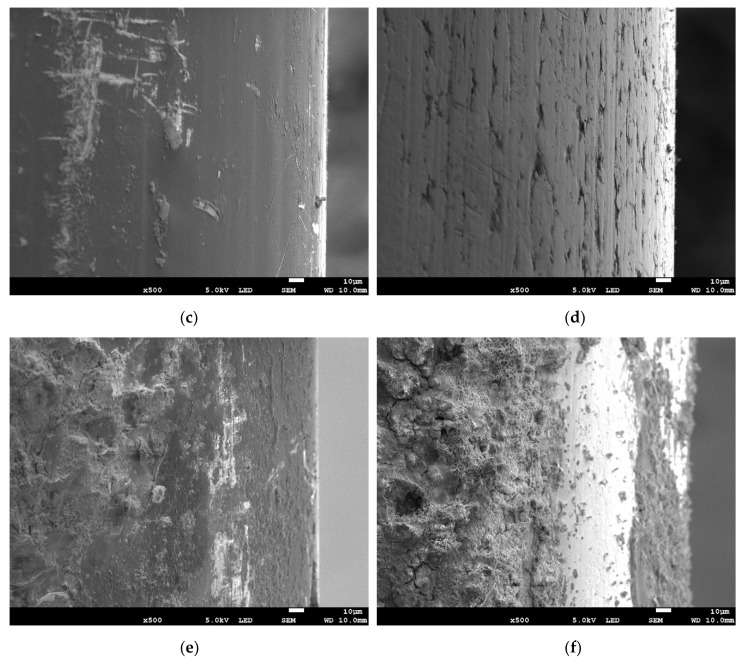
Surfaces of flux-cored wires: (**a**) A wire in delivery condition; (**b**) B wire in delivery condition; (**c**) A wire stored in GWS; (**d**) B wire stored in GWS; (**e**) A wire stored in MAB; (**f**) B wire stored in MAB.

**Figure 6:**
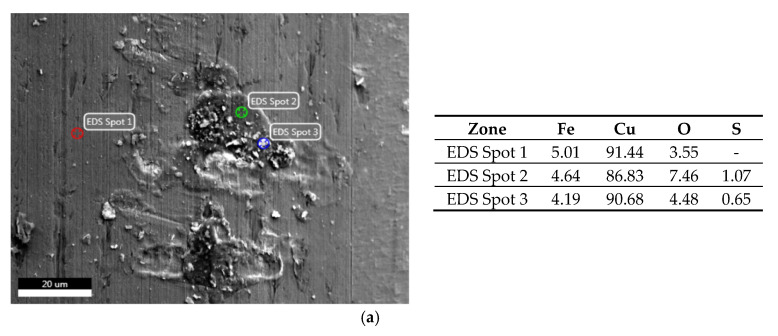

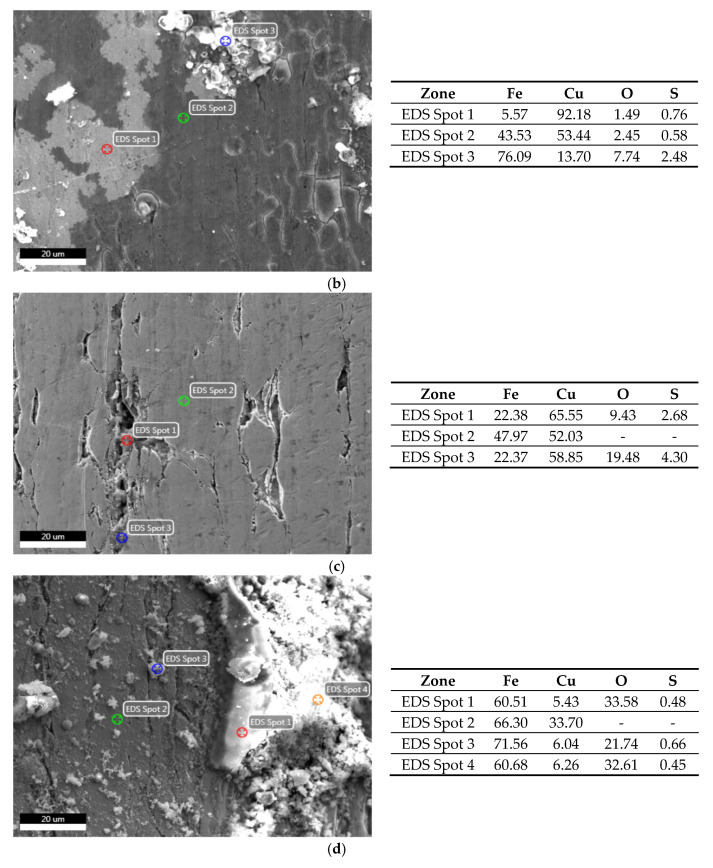
SEM image and spot EDS analysis of flux-cored wires: (**a**) A wire stored in GWS; (**b**) A wire stored in MAB; (**c**) B wire stored in GWS; (**d**) B wire stored in MAB.

SEM images show that the surfaces of both wires in delivery condition significantly differ (Figure 5a,b). The A wire is smooth, has a homogeneous surface without gaps in the continuity of the copper coating. On the contrary, B wire shows longitudinal grooves on the surface, located parallel to the axis of the wire and the direction of material forming. After storing the wires in GWS, A wire still has a smooth surface; only small precipitations can be observed on it (Figure 5c). The EDS analysis (Figure 6a) shows that these products mainly contain copper. B wire behaved similarly—in the grooves visible in Figure 5d, copper compounds accumulated (Figure 6c). Both wires from the MAB location are characterized by a high degree of degradation (Figure 5e,f). On their surface, precipitations of iron oxides were found, which was confirmed by the EDS analysis (Figure 6b,d). The differences between these wires can be seen in the SEM photos at higher magnification—A wire is only locally covered with a thicker layer of corrosion products, but a thin, brittle layer of iron oxides has formed on its surface (Figure 6b). A much thicker layer of corrosion products has accumulated on B wire. Figure 5e,f show the most degraded parts of the wires. Figure 5c–f also show the edge of the wires, which in each case shows no signs of corrosion other than atmospheric corrosion.

Tensile test of base material—S355J2 steel was carried out to find the basic mechanical properties. The mean tensile strength of the material is 581 MPa with standard deviation of 1.75 MPa. The elongation of base material is 35% with standard deviation of 2.8%.

**Figure 7:**
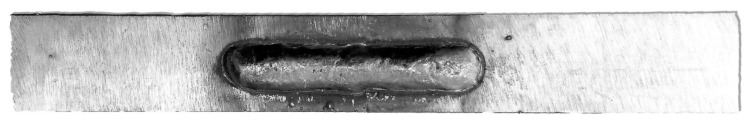
Macroscopic view of the bead-on-plate weld.

The wires were used to make bead-on-plate welds. The welds were straight, smooth, and did not show excessive spatter, surface irregularity, porosity, cracks or undercuts. An example of specimens with weld bead is shown in Figure 7. They were all made with the use of the same parameters (Section 2) and their appearance did not significantly differ—they were all acceptable from the point of view of quality standards.

**Figure 8:**
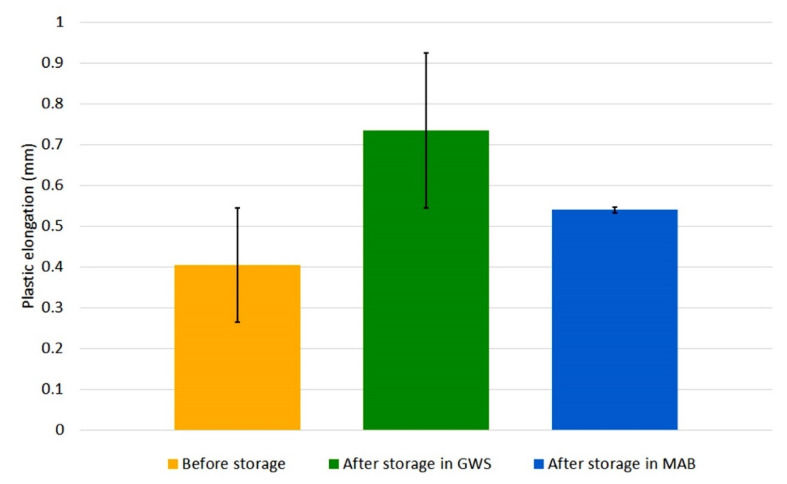
Change in plastic elongation of welds made with A wire, caused by storage for 1 month.

**Figure 9:**
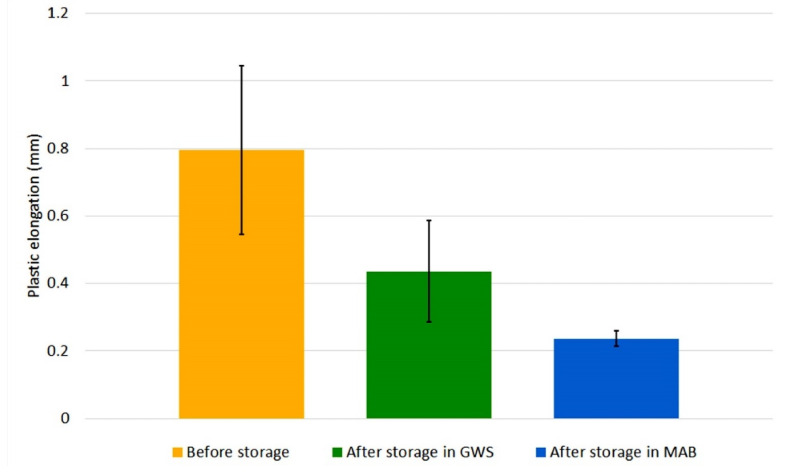
Change in plastic elongation of welds made with B wire, caused by storage for 1 month.

The specimens with bead-on-plate welds were subjected to a static tensile test. Earlier tests of wires stored in industrial conditions have shown an increase in tensile strength of deposited metal after storage of wires [20]. In order to determine the effect of temperature and relative humidity on plasticity of bead-on-plate welds, the elongation value was read from stress–strain curves and plasticity index (*E_r_*) was calculated. Figure 8 and Figure 9 show the change in elongation of welds made by the tested welding wires due to their storage (error bars indicate standard deviation).

The obtained values of plastic elongation and standard deviation for all specimens confirm the statistical significance of the results. Although the wires have the same specification, their elongation values in the initial state differ. The A wire, regardless of its storage location, was characterized by an increase in plastic elongation. The increase was greater in the case of GWS—nearly twice. However, B wire showed a different trend. Each of the specimens, welded with both GWS and MAB wire, had a smaller plastic elongation compared to the specimen welded with the wire in delivery condition. A large drop was particularly visible in the wire from MAB—the plasticity of the weld metal dropped almost four times with a double drop for the wire stored in GWS.

**Figure 10:**
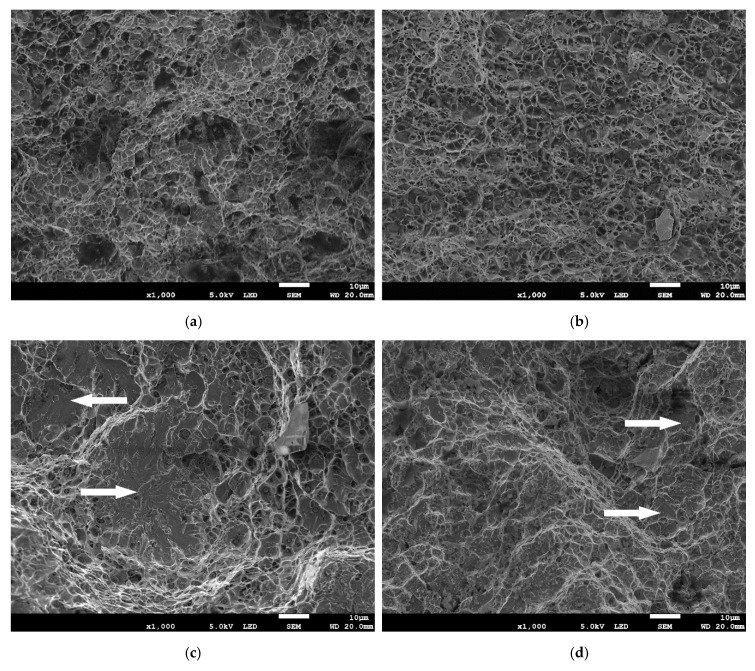

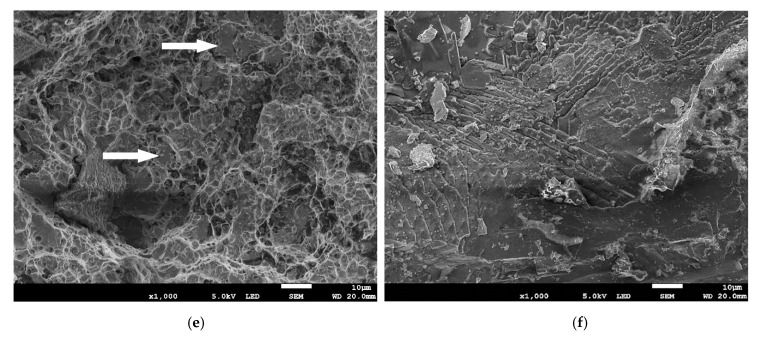
Fracture surfaces of specimens after tensile tests: (**a**) specimen made using A wire in delivery condition; (**b**) specimen made using B wire in delivery condition; (**c**) specimen made using A wire stored in GWS; (**d**) specimen made using B wire stored in GWS; (**e**) specimen made using A wire stored in MAB; (**f**) specimen made using B wire stored in MAB.

Figure 10 shows SEM images of fractures after tensile test. In Figure 10a,b, dimples, characteristic of the ductile microvoid coalescence fracture, are clearly visible. Such a fracture mode predominated in specimens made with wires in delivery condition. An increased share of the brittle fracture (marked with white arrows) was observed in specimens made of wire from the GWS and in the specimen made of A wire from MAB (Figure 10c–e). In this case, the quasi cleavage fracture was found. Three zones can be observed—at the face the fracture was more ductile, dimples appeared larger, in the middle zone between dimples flat cleavage areas appeared and at the root there were dimple clusters and larger areas of cleavage, with large steps. Figure 10f shows the fracture surface of a specimen made using B wire stored in MAB. In this specimen, cleavage fracture dominates. At 1000× magnification, only single dimples are visible, situated between large cleavage areas.

Results of diffusible hydrogen content in deposited metal are shown in Table 4. The mean value of diffusible hydrogen obtained from wires before storage were 3.46 and 4.36 for A and B wire, respectively. For both wires the storage caused the increase in diffusible hydrogen content. The highest diffusible hydrogen amount was collected for B wire stored in MAB.

## 4. Discussion

The differences in recorded temperatures for studied areas result from both geographical and anthropogenic factors. The north-eastern part of Poland is an area where the continental climate with a higher temperature gradient begins to dominate. It results in clearly lower temperatures compared to Gdańsk, which is a port city, with a predominance of maritime climate. The second factor results from the degree of urbanization of both locations—Mońki is a small city and Gdańsk is a main city of a metropolitan area in northern Poland. The more specifically analyzed conditions in which the wires were stored may lead to the reason for differences in recorded relative humidity. The welding side is usually a drier place, there are not many sources of moisture. In contrast, unheated MAB may be exposed to increased air flow and water vapor supply from the environment. This analysis of the storage conditions is visible in Figure 2 and Figure 3.

Comparison of these results with information from flux-cored wire manufacturers clearly shows that the conditions for storage of wires in MAB do not fall within the “safe” ranges declared by the manufacturers. At the same time, the GWS was a theoretically safe place—the conditions should not cause changes in the appearance of the wires and the weld properties.

The degradation of the surface of wires is one of the first signs of environmental impact on the material. General and atmospheric corrosion develops on the surface of the wires [5,6,26]. Depending on the storage location, it had a different intensity—A and B wires in MAB were exposed to increased relative humidity, which was the reason for accelerating surface degradation. High temperature may also accelerate the development of corrosion, however, in the studied locations it did not deviate from safe values. This suggests that under the studied conditions, relative humidity was a factor of greater importance in the case of wire surface degradation. No signs of degradation on the surface of the wires stored in the GWS suggests that there were no processes that could impair the weld metal. This was verified by conducting static tensile tests of bead-on-plate welds made using those wires.

The results of the base material tensile tests show a high similarity of the tensile strength values of the base material and investigated wires. This indicates also the correct selection of welding consumables for the process. It is worth noting that comparing these results with tensile test results obtained with the use of modified specimens as in current study and in [20] have to be done carefully because the specimens have a different shape (dimensions and notch) and a different structure (weld). 

Observation of the bead-on-plate weld surfaces was a valuable analysis—the proper appearance of joints is one of the basic quality criteria [43]. Each of the wires, despite the differences in surface degradation, allowed us to obtain sound welds. No visible imperfections suggest that the stability of arc glow was sufficient. For real-working welding process, the appearance of the weld and sufficient stability are often the criteria confirming the suitability of the stored wire for welding. In the case of flux-cored wires, this can be a particularly dangerous simplification.

The plasticity of the welds was influenced by the storage conditions of the tested flux-cored wires. The A wire is clearly less sensitive to the environment. In this case, changes occurred in the direction of increasing plasticity, but they were less intense than in the case of B wire. The degradation of B wire caused a deterioration in the plasticity of bead-on-plate welds in both storage environments. The list of plasticity indexes values (Table 5) shows very large differences which were found for the tested conditions.

A change in plasticity can also be observed by comparing the mode of fracture of the tensile tested specimens (Figure 10). The most characteristic is the transition from the ductile microvoid coalescence fracture to the almost complete cleavage fracture for the specimen made using B wire stored in MAB. Such a great change is directly related to the results of plasticity indexes (Table 5), which also indicate a very intense degradation of bead-on-plate weld plasticity for this wire and location. In all fracture surfaces, at least some dimples were found and they were all equiaxed, which confirms that tensile loading was the main loading type responsible for breaking of specimens.

The research on the diffusible hydrogen content proved that the wire storage influences its amount in weld metal. Each of the tested wires was characterized in the initial state by H5 level—the content of diffusible hydrogen in deposited metal did not exceed 5 mL/100 g. The A wire showed relatively good resistance to 1 month storage under the tested conditions. The mean value of diffusible hydrogen content increased slightly, but still did not exceed H5 level. For B wire stored under the same conditions, the diffusible hydrogen content changed significantly. The GWS conditions, although described by the recommended levels of temperature and relative humidity, caused an almost twofold increase in diffusible hydrogen content and it reached the level of H10 (between 5 and 10 mL/100 g). Even greater changes took place in the conditions of the MAB. The diffusible hydrogen content exceeded the H10 level and so the wire would have to be described as high hydrogen consumable. Such a high level of diffusible hydrogen disqualifies consumables from welding of responsible structures, including marine and offshore applications.

Literature research indicates that the reduction in plasticity of steel can be caused by various factors—changes in structure, the presence of notch, hydrogen or contamination [29,44,45]. Structural changes of the tested specimens resulting from the welding heat could change the plasticity of the material in relation to the base material, however, by comparing individual specimens with each other, this factor can be eliminated because all the specimens were characterized by the same dendritic structure, typical for welds. The second factor—the presence of a notch also had an effect on the material’s properties relative to the base material. However, due to the presence of the same notch in all specimens (resulting from their design), this is not a factor affecting the differences in the plasticity indexes from Table 5. In addition, the welding process was carried out each time on the same device, using the same parameters, and the penetration depth for a given type of wire was constant thanks to the use of a robotic station. The bead-on-plate welds lengths were always the same, so that the thermal conditions around the notch were always the same.

The factors that could have the greatest impact on recorded changes in plasticity are the presence of hydrogen and contamination in the bead-on-plate weld material. Their sources could be: moisture, hydrogen that penetrated the wires, contaminations deposited on the surface of the wire, contaminations being inside the wire and corrosion present on the surface of the wire. One of the signs of the presence of hydrogen in steel is a decrease in its plasticity [44,45]. This effect was recorded for B wire in both locations. A significant decrease in plasticity was observed in this wire, and at the same time the measurements of the diffusible hydrogen content confirmed its increased level. A larger drop in MAB may be the result of more extreme relative humidity conditions, which were conducive to both the penetration of hydrogen and the development of corrosion on the surface of the wire.

The observed increase in the plasticity index for A wire suggests that in this case the environment had much less impact on the stored wire. Despite the fact that both wires are seamless and copper plated, a smaller amount of contaminations was observed on the surface of A wire, both after storage in the GWS and in the MAB. At the same time, when comparing the surfaces of both wires in the delivery condition, significant differences in the structure of the wires can be observed— A wireis smoother, has no gaps or grooves on the surface, which better protects it against the destructive influence of the environment. 

It should be added that only specimens made with the same type of wire can be directly compared. Despite maintaining the same welding parameters, differences in the structure of wires (e.g., filling rate) result in, among other consequences, different welding current density values. Therefore, bead-on-plate welds differing in penetration depth, characteristic for each of the wires, are obtained and for comparison, determination of the notch size as the factor influencing the results for analysis is required.

The results indicate that storage of flux-cored wires in real conditions, more extreme than the manufacturer’s recommendations, will cause changes in the bead-on-plate weld plasticity expressed by the plasticity index. The changes observed and presented in this article do not show one direction, they depend on the chemical composition of the wire, its structure and the conditions in which the wire was stored. However, the storage period of just one month is sufficient for the environmental impact on the plasticity of bead-on-plate welds to be significant.

## 5. Conclusions

Research was focused on assessing the impact of storage conditions in extremely different geographical and industrial locations of two types of flux-cored wires on plasticity of bead-on-plate welds. Based on the findings of the study, the following conclusions were drawn:

1. Under the influence of the environment, the appearance of the wires clearly changed, a layer of corrosion product was formed on wire surfaces. The plastic properties of bead-on-plate welds made using those wires were also changed.
2. The wires stored in the welding site had a less degraded surface and after storage the copper compounds appeared in grooves. After being stored in an agricultural building, the surface of the wires was covered with iron compounds—for the B wire the amount of corrosion products is greater.
3. The presented results indicate that even a relatively short, monthly storage of flux-cored wire can contribute to a nearly fourfold decrease in the value of the plasticity index of bead-on-plate welds in the intrinsic hydrogenation conditions. At the same time, the second of the tested wires showed a slight increase in the value of the plasticity index in both tested environments.
4. The mode of the fracture in tensile specimens varied depending on the storage conditions and grade of wire. Under the influence of monthly storage, there was a change from the ductile microvoid coalescence fracture to the almost complete cleavage fracture.
5. Storage of flux-cored wires may affect the diffusible hydrogen content in deposited metal and increase its amount to over 10 mL/100 g.
6. In the case of non-alloyed flux-cored wires, the assumption that only surface appearance and arc stability are enough criteria to accept wires for welding can be a dangerous and misleading simplification.
7. Taking all things into consideration, it can be assumed that the type of material affects the direction of plasticity changes, while location affects the intensity of plasticity changes.

## Figures and Tables

**Table 1:** Chemical composition in percentage by weight (wt.%) of the base material and weld metal made with the use of wires according manufacturers certificates.

	C	Si	Mn	P	S	Cu	Fe
S355J2	0.19	0.22	1.36	0.017	0.015	0.21	bal.
A wire	0.05	0.57	1.42	0.013	0.006	0.29	bal.
B wire	0.05	0.49	1.38	0.010	0.008	-	bal.

**Table 2:** Mechanical properties of the weld metal made with the use of wires according to the manufacturer’s certificates.

	Tensile Strength Rm (MPa)	Yield Strength Rp (MPa)	Elongation A (%)
A wire	605	539	22
B wire	550	460	24

**Table 3:** Values of statistical measures and coefficients describing changes in storage conditions.

Condition/Location	Temperature (°C)		Relative Humidity (%)	
	GWS	MAB	GWS	MAB
Min	18.4	-2.2	20.3	43.2
Max	21.9	13.5	44.0	100.0
Range	3.5	15.7	24.6	56.8
Mean	19.9	5.4	34.8	85.0
Standard deviation	0.7	3.1	4.8	12.1
Coefficient of variation	3.3	58.1	13.7	14.3

**Table 4:** Diffusible hydrogen content in deposited metal (mercury method) obtained using flux-cored wires.

Storage Condition	Diffusible Hydrogen Measurements (mL/100 g)			
	A wire		B wire	
	H_D_	Standard Deviation	H_D_	Standard Deviation
Before storage	3.46	0.58	4.36	0.35
GWS	4.41	0.16	8.58	1.21
MAB	4.05	0.79	10.13	0.88

**Table 5:** Plasticity indexes for bead-on-plate welds made using A and B wires after storage.

Location	A Wire	B Wire
GWS	1.82	0.55
MAB	1.34	0.30
